# Street food in Eastern Europe: a perspective from an urban environment in Moldova

**DOI:** 10.1017/S0007114520002020

**Published:** 2020-11-28

**Authors:** Gabriela Albuquerque, Marcello Gelormini, Inês Lança de Morais, Sofia Sousa, Susana Casal, Olívia Pinho, Pedro Moreira, João Breda, Nuno Lunet, Patrícia Padrão

**Affiliations:** 1 EPIUnit – Instituto de Saúde Pública, Universidade do Porto, Rua das Taipas 135, 4050-600 Porto, Portugal; 2 Nutrition, Physical Activity and Obesity Programme, Division of Noncommunicable Diseases and Life-Course, WHO Regional Office for Europe, UN City, Marmorvej 51, 2100 Copenhagen, Denmark; 3 Faculdade de Ciências da Nutrição e Alimentação da Universidade do Porto, Rua Dr. Roberto Frias, 4200-465 Porto, Portugal; 4 Laboratório Associado para a Química Verde (LAQV/REQUIMTE), Laboratório de Bromatologia e Hidrologia, Faculdade de Farmácia, Universidade do Porto, Rua D. Manuel II, Apartado 55142, Porto, Portugal; 5 Centro de Investigação em Atividade Física, Saúde e Lazer, Universidade do Porto, Rua Dr. Plácido Costa, 4200-450 Porto, Portugal; 6 WHO European Office for the Prevention and Control of Noncommunicable Diseases, WHO Regional Office for Europe, Leontyevsky Pereulok, 125009 Moscow, Russian Federation; 7 Departamento de Ciências da Saúde Pública e Forenses e Educação Médica, Faculdade de Medicina da Universidade do Porto, Alameda Prof. Hernâni Monteiro, 4200-319 Porto, Portugal

**Keywords:** Eastern Europe, Food processing, Nutritional value, Ready-prepared foods, Street food

## Abstract

Street food is popular in Eastern Europe, but its diversity and nutritional value are unknown. This study aimed to characterise the street food environment in Chişinău, Moldova, including the vending sites and vendors, food availability and nutritional composition of foods and beverages. All street food vending sites (single point of sale) located in a 1-km buffer centred on the main public market were systematically selected (*n* 439; *n* 328 participants). Data on vending sites’ characteristics (mobility, type of physical set-up and access to electricity), operating periods and food availability were collected. Samples of the most commonly available foods of unknown composition were collected (twenty-eight home-made and twenty-four industrial). Macronutrients, Na and K were quantified through chemical analysis. Fruits, beverages and food other than fruits were available in 2·5, 74·3 and 80·8 % of the vending sites, respectively. Among the latter, 66·4 % sold only industrial foods (e.g. pretzels, biscuits, wafers, chocolate and ice cream), 21·5 % only home-made (e.g. savoury and sweet pastries) and 12·1 % both. Home-made foods presented larger serving sizes and energy/serving (median kJ/serving: 1312·5 *v.* 670·3, *P* = 0·022); industrial foods were more energy-dense (median kJ/100 g: 1797·0 *v.* 1269·8, *P* = 0·002). High SFA, *trans*-fat and Na contents were found, reaching 10·9 g/serving, 1·4 g/serving and 773·7 mg/serving, respectively. Soft drinks and alcoholic beverages were available in 80·7 and 42·0 % of the vending sites selling beverages, respectively. Concluding, industrial snacks and home-made pastries high in Na and unhealthy fat were frequent in Chişinău. Prevention of diet-related diseases in Moldova may benefit from the improvement of the nutritional profile of street food.

Eastern Europe is currently facing nutrition transition. Over the last three decades, there has been an increase in energy availability, mostly due to increased availability of animal, fat, sugar and high-protein foods^(1,2)^. High consumption of red and processed meat, contrasting with low consumption of fruits, vegetables, legumes, whole grains, nuts and seeds, has also been described^(3)^. A concurrent increase in the burden of non-communicable diseases (NCD) has been reported^(2,3)^, which is a well-described epidemiological characteristic of nutrition transition^(4)^. In Moldova, overweight and obesity show an increasing trend; the most recent estimates indicate a prevalence of approximately 20·0 % of obesity among adults. CVD, cancer and other NCD are the leading causes of death (59·0, 15·0 and 16·0 %, respectively)^(5)^.

Typical socio-economic changes in transitional societies include an increase in the urban working force, particularly among women, which is in turn associated with increased consumption of food away from home^(6)^, such as street food. The definition of street food was proposed by the FAO and the WHO, as ‘ready-to-eat foods and beverages prepared and/or sold by vendors or hawkers especially in the streets and other similar places’^(7,8)^. The convenience and affordability^(9,10)^ make street food accessible, frequently replacing home meals^(10,11)^. In Eastern European countries, it is part of the cultural heritage and gastronomy, reflecting the availability of local food products, culinary practices, lifestyles and consumer preferences^(12,13)^.

Current literature on street food is mainly focused on food safety, disregarding topics such as the types of foods available and their nutritional composition^(10,14,15)^. Although a few studies have estimated that street food largely contributes to the total intake of fat, *trans*-fatty acids (TFA), salt and sugar^(10)^, to our knowledge, no research on street food has been conducted in this world region. Furthermore, Moldova is one of the Eastern European countries where nationally representative data on food availability and dietary habits are scarce^(16)^. The aim of this study was to characterise the street food environment in Chişinău, the capital city of Moldova, including the vending places and food availability, as well as the nutritional composition of the most commonly available foods and beverages.

## Methods

This study was implemented within the scope of the FEEDcities project, supported by the WHO – Europe, which used a stepwise standardised methodology to characterise the street food environment in countries in Central Asia and Eastern Europe^(17)^. In this study, specifically, step 1 comprised identification of street food vending sites and characterisation of vending sites, vendors and products available, by direct observation and by invitation of street food vendors to answer additional questions. Step 2 comprised collection of samples of the most frequently available foods for chemical analysis.

### Setting and study design

This cross-sectional evaluation was conducted between June and August 2016 in Chişinău, the capital city of Moldova. Moldova is a lower-middle-income country, with an estimated urbanisation rate of 42·5 % in 2016^(18)^. Approximately 690 000 individuals were living in Chişinău, corresponding to 20 % of the whole country population (2·8 million in 2016)^(19)^.

In a site visit conducted for preparing fieldwork, previous to data collection, members of the research team observed that in this city, most street food vendors were concentrated in the central market and its surroundings, in the *Centru* sector, the central business district. To define the study area, a 1-km diameter buffer was built around the central market (with the centroid in its geographic midpoint), covering it and its surroundings. To define the sample size, it was assumed, for a design effect up to 1·5, that a sample size of approximately 300 vending places would yield 95 % CI up to 15 % wide for observed proportions ranging between approximately 30 and 70 %, and 95 % CI for means with a width of approximately 30 % of the observed standard deviation.

Eligible vending sites (single point of sale regardless of whether one or more vendors were operating) were defined as the business establishments selling ready-to-eat food, including beverages and/or snacks. This included mobile vendors, as well as sellers with semi-static or stationary vending units. The exclusion criteria were the following: (1) food establishments with four permanent walls; (2) permanent storefront businesses; (3) street vendors selling exclusively non-food products or raw foods not ready-to-eat and (4) food stalls and carts which were part of permanent stores or licensed establishments.

### Data collection: vending sites, vendors and food availability

The study area was systematically assessed in nine consecutive working days, between 27 June and 6 July. After registering the global positioning system (GPS) coordinates of each vending site, ten interviewers collected the following information, through direct observation: sex of the vendor, mobility of the vending site and type of physical set-up. Stationary vending sites were further classified into formal (stand (an upright structure where street food is prepared and/or displayed), *dukoni* (restaurant serving traditional fast food selling directly on the street through an open window), kiosk (small open-fronted hut or cubicle where typically newspapers are sold, as well as a sort of foods and beverages), *kvass* barrel (vending site consisting of a portable metal barrel containing *kvass*, a traditional fermented beverage made from rye bread, supported by an upright structure), table with chairs for customers (displayed in the public street and not being part of a permanent storefront business) or truck (large motorised vehicle, such as a van or trailer, equipped to cook, prepare, serve and/or sell food)) or informal (bench with table, pushcart and other improvised sites as freezers, refrigerators or coolers) (online Supplementary Table S3).

Afterwards, interviewers approached the vendor to explain the study objectives and procedures and to ask for express consent to participate in the study. When the vendor agreed, the interviewers carried out computer-assisted personal interviews, enquiring about vending site ownership and food availability, including products sold and serving sizes. Given the limited time of food vendors operating mobile vending sites to answer the questionnaire, additional questions regarding vending sites’ characteristics (access to electricity) and food vending activity (operating periods – during the week; during the year and under which weather conditions) were asked only to vendors operating on stationary vending sites. Additional information is available on the protocol of this study, including the structure of the questionnaire^(17)^.

A total of 439 eligible vending sites were identified in the study area, and all vendors were invited to participate; 328 (74·7 %) vendors accepted. No statistically significant differences were found between participant and non-participant vendors regarding the type of vending site they were operating (online Supplementary Table S1).

Foods available were grouped according to their nature, into fruit (product *in natura*, either fresh or dry), beverages (any alcoholic and non-alcoholic drink) or food other than fruit. According to FAO standards^(11)^, food other than fruit was further classified as home-made (self-prepared food products, at home or on the street, even if using industrial ingredients) or industrial (commercially prepared food products sold by street vendors without further preparation and/or cooking, encompassing both processed and unprocessed foods^(20)^). Home-made food was also grouped in ‘cooked’, ‘prepared but uncooked’ and ‘uncooked and unprepared’. Beverages were further classified into soft drinks, water, fruit juice-based drinks, fresh fruit juice-based drinks, milk, milk-based drinks, alcoholic beverages, energy drinks, coffee, tea or traditional beverages.

### Food sample collection

Following the computation of the frequency of each of the identified foods and beverages across all the vending sites included in the study (*n* 328), samples of the most commonly available foods and beverages of unknown composition, that is, for which there was an absence of standardised information on nutritional composition, including nutrition labels or food composition tables, were collected for nutritional composition assessment. The most frequent home-made foods (*n* 7) were savoury *placintă* (*placintă*: fried pastry, usually made from dough made of flour, water and sunflower oil, filled with cheese, vegetables, meat or potatoes or sweet fillings, usually served in a pie shape, but may have various shapes), sausage roll, *pateuri* (traditional fried pastry made from leavened dough filled with cheese, vegetables or meat, usually an oval shape and served in individual portions), sweet roll/*chiflă* (sweet bun made of leavened dough, either flaky or brioche type, usually filled with a chocolate, vanilla or cherry cream), *cheburec* (traditional fried savoury pastry generally filled with meat such as chicken or beef), sweet *placintă* and sweet doughnut. The most frequent industrial foods (*n* 6) were biscuits, chocolates, candies, chewing gums, croutons and sunflower seeds *in natura*, but, given that the expected nutritional composition of candies, chewing gums and sunflower seeds *in natura* does not vary widely, the following most available industrial foods were selected: wafers, ice cream and pretzels. A local industrial beverage, *kvass*, was additionally selected for collection, given its full availability in this setting and unknown composition. Four samples of each of these items (hereafter referred to as ‘food’, although this beverage was included) were collected from different vending sites. For one food (*cheburec*), it was only possible to collect two samples, and for two other foods (*plăcintă* and *pateuri*), five samples were purchased. A total of fifty-two samples (twenty-eight home-made and twenty-four industrial) were collected. Online Supplementary Table S4 displays one example of each of the street foods collected, along with a brief description for the ‘local foods’.

The definition of the vending sites where the food samples were collected was carried out according to a standardised procedure: in each day, ten coordinates were randomly selected from the list of the GPS coordinates of the eligible vending sites previously assessed. A sample of each food product, corresponding to one serving, was bought whenever possible at these vending sites. If not possible, a systematic selection procedure was followed, in which field researchers start moving north from that point and change direction clockwise (first east, then south, then west, then north again) whenever the limits of the study area or a physical barrier (such as a wall or a canal) are met, until reaching vending sites where these foods were available. In each vending site, only one sample of home-made or industrial food was obtained. The fifty-two food samples were collected in seven consecutive days.

### Nutritional composition assessment

After collection, samples were homogenised, weighed and stored in a freezer (−18°C) until the nutritional composition assessment, which included the analysis of (a) moisture, by oven drying at 103°C until constant weight; (b) protein, by the Kjedahl method with a conversion factor of 6·25; (c) fat, by the Soxhlet method, (d) total minerals by dry ashing at 500°C, (e) total carbohydrates plus fibre by difference and (f) energy value by the Atwater general factors, all following the standard Association of Analytical Communities^(21)^ routine analysis in the Laboratory of Bromatology of the University of Porto (LAQV/REQUIMTE), a partner in the FEEDcities project^(17)^. For the analysis of the fatty acid composition, fat was extracted separately with organic solvents followed by cold transesterification^(22)^. Separation of the methyl esters was achieved by GC with flame ionisation detection, using a column specific for *cis*/*trans* separation (Agilent J&W Select FAME, 50 m) and reference standards for calibration (Supelco). The results are expressed on a relative fatty acids basis and grouped as SFA, MUFA, PUFA, *n*-3 and *n*-6 fatty acids (TFA). Na and K were analysed following a validated flame photometric method^(23)^. The analytical results were the average of two determinations per food sample. A third determination was conducted only when the first two were not in agreement; in this case, the average of the two concordant results was calculated. All the analytical results were expressed by serving size, in g.

### Statistical analysis

The vending sites and their food availability were characterised through absolute and relative frequencies (categorical variables). Pearson’s *χ* ^2^ and Fisher’s exact tests were used to identify statistically significant differences between stationary and mobile vending sites and between formal and informal stationary vending sites.

Regarding the nutritional composition assessment, mean serving sizes per food, in g, were calculated as the mean weight of the four individual samples collected for each of the foods. Likewise, per-serving levels of each nutrient were calculated as the mean content of the four individual samples and expressed in g/serving (macronutrients) or mg/serving (micronutrients). Results were presented for each food, as the mean and range of energy (kJ), carbohydrates, protein, water, total fat, including SFA, MUFA, PUFA, *n*-3 fatty acids, *n*-6 fatty acids, TFA, Na and K per serving and molar Na:K ratios. To convert energy in kJ to kcal, the values should be divided by 4·184. To calculate individual molar Na:K ratios, contents of Na and K of each sample were converted into mmol based on their molar weights, 23·0 g/mol and 39·1 g/mol, respectively^(24)^. The energy content of home-made and industrial foods was compared using the non-parametric Mann–Whitney *U* test. A critical level of significance (*P*) <0·05 was considered statistically significant.

Statistical analysis was performed using the software STATA® version 11.0 for Windows®.

### Ethical considerations

This study was conducted according to the Declaration of Helsinki, and all procedures involving human subjects were approved by the Ethics Committee of the Institute of Public Health of the University of Porto (CE16058). Verbal informed consent was obtained from all vendors.

## Results

### Food vendors and vending sites

Over four in every five street vendors were women (88·1 %), and a small proportion were owners (10·4 %), except in mobile vending sites, where most vendors (71·4 %) were owners of the business (Table 1).

**Table 1.** Characteristics of street food vending sites and street food availability, by type of vending site in Chişinău, Moldova (*n* 328) (Numbers and percentages)

			Type of vending site				*P*
	Total (*n* 328)		Stationary (*n* 314)		Mobile (*n* 14)		
	*n*	%	*n*	%	*n*	%	
Vending sites characteristics							
Food vendor sex (women)	289	88·1	280	89·2	9	64·3	0·005*
Food vendor ownership	34	10·4	24	7·6	10	71·4	<0·001*
Access to drinking water	317	96·7	303	96·5	14	100·0	0·476
Access to toilet facility	318	97·0	306	97·5	12	85·7	0·012*
Food availability							
Fruit	8	2·5	8	2·6	0	0·0	1·000
Food other than fruit	265	80·8	252	80·3	13	92·9	0·242
Industrial	176	66·4	173	68·7	3	23·1	<0·001†
Home-made and industrial	32	12·1	31	12·3	1	7·7	
Home-made	57	21·5	48	19·1	9	69·2	
Home-made cooked‡	87	97·8	77	97·5	10	100·0	1·000
Home-made prepared but not-cooked‡	21	23·6	21	26·6	0	0·0	0·109
Home-made non-prepared and non-cooked‡	1	1·1	1	1·3	0	0·0	1·000
Beverages§	243	74·3	240	76·7	3	21·4	<0·001†
Soft drinks	196	80·7	194	80·8	2	66·7	0·477
Water	187	77·0	186	77·5	1	33·3	0·134
Fruit juice-based drinks	140	57·6	139	57·9	1	33·3	0·576
Alcoholic beverages‖	102	42·0	102	42·5	0	0·0	0·266
Coffee	79	32·5	76	31·7	3	100·0	0·033†
Energy drinks	76	31·3	76	31·7	0	0·0	0·554
Tea	69	28·4	66	27·5	3	100·0	0·022†
Fresh fruit juice-based drinks	17	7·0	17	7·1	0	0·0	1·000
Milk	6	2·5	6	2·5	0	0·0	1·000
Milk-based drinks¶	3	1·2	3	1·3	0	0·0	1·000
Traditional dairy beverages**	2	0·8	2	0·8	0	0·0	1·000

* Statistically significant differences according to Pearson’s *χ*^2^ test, for a confidence level of 95 % (*P* < 0·05).

† Statistically significant differences according to Fisher’s exact test, for a confidence level of 95 % (*P* < 0·05).

‡ Sum of the values for this variable is higher than the total number of home-made foods, as each vendor could have available foods prepared in different ways.

§ Sample size for these variables is smaller, due to missing data (*n* 313).

‖ Alcoholic beverages: beer (*n* 60), *kvass* (*n* 45), alcopop (*n* 7), distilled beverages (*n* 3) and wine (*n* 2).

¶ Milk-based drinks: milk drinks and cocktails (*n* 1) and hot chocolate (*n* 2).

** Traditional dairy beverages: *kephyr* (*n* 1) and *yogurt* (*n* 1).

Most vending sites were located inside the market (online Supplementary Fig. 1) and were stationary (95·7 %), of which the most frequent physical set-ups were stands (35·4 %) or kiosks (29·6 %) (online Supplementary Table S1). Most stationary vending sites had access to electricity (88·9 %), drinking water (96·5 %) and toilet facilities (97·5 %) and were operating 7 d/week (87·3 %), all year long (79·9 %) and under every type of weather (81·2 %). The informal vending sites were less likely to be operating the whole year (43·1 *v*. 87·1 %, *P* < 0·001) and under every type of weather (56·9 *v*. 85·9 %, *P* < 0·001) (online Supplementary Table S2).

### Food availability

Fruit and beverages were sold, respectively, in 2·5 and 74·3 % of the vending sites. Food other than fruit was sold in 80·8 % of the vending sites, of which 66·4 % sold only industrial foods (e.g. pretzels, biscuits, wafers, chocolate and ice cream), 21·5 % only home-made (e.g. savoury – *cheburec*, *pateuri*, *plăcintă*, sausage roll – and sweet pastries – *plăcintă*, *chiflă* and doughnut) and 12·1 % both home-made and industrial foods. The mobile vending sites were more likely to sell only home-made foods (69·2 *v*. 19·1 %, *P* < 0·001) and less likely to sell only industrial foods (23·1 *v*. 68·7 %, *P* < 0·001). Most home-made foods were cooked (97·8 %). Stationary vending sites were the only ones selling fruit, and where beverages were more frequently (76·7 *v*. 21·4 % in mobile vending sites, *P* < 0·001). The most commonly available beverages were soft drinks (80·7 %), water (77·0 %), fruit juice-based drinks (57·6 %) and alcoholic beverages (42·0 %) (Table 1).

### Nutritional composition

Industrial foods were more energy-dense than home-made foods (median kJ/100 g: 1799·1 *v*. 1271·9, *P* = 0·002), but the latter presented higher energy content per serving (median kJ/serving: 1312·5 *v*. 670·3, *P* = 0·022). The mean energy content per serving was highest in industrial and home-made sweet snacks: wafers (1909·6 kJ/serving) and sweet *chiflă* (1809·2 kJ/serving).

*Kvass*, a traditional low-alcohol fermented beverage, presented the lowest content in every macronutrient. The foods with the highest average protein content were home-made savoury pastries: sausage roll (11·9 g/serving) and savoury *plăcintă* (10·1 g/serving). Industrial wafers (22·4 g/serving) and home-made *pateuri* (18·3 g/serving) showed the highest mean fat content and industrial chocolate and chocolate candy (63·2 g/serving) and home-made sweet *chiflă* (61·4 g/serving) the highest mean carbohydrates’ content (Table 2).

**Table 2.** Nutritional composition (energy and macronutrients) of the street food samples evaluated by laboratorial analysis, per serving (Mean values and ranges)

		Serving size (g/serving)		Energy (kJ/serving)*		Protein (g/serving)		Carbohydrates (g/serving)		Total fat (g/serving)		Water (g/serving)	
	*n*	Mean	Min–max	Mean	Min–max	Mean	Min–max	Mean	Min–max	Mean	Min–max	Mean	Min–max
Industrial food													
Biscuits	4	33	33–33	696·4	663·0–725·5	2·0	1·4–2·8	21·9	21·3–22·5	7·9	6·7–8·8	1·2	0·6–1·8
Chocolate	4	88	82–98	1787·6	1683·9–1990·8	5·0	1·0–9·8	63·2	44·1–83·3	17·1	9·2–30·6	1·9	0·6–3·7
Ice cream	4	56	33–71	624·7	424·0–886·8	2·1	1·2–2·9	18·3	7·4–26·5	7·5	3·7–10·5	27·3	16·2–31·9
*Kvass*	4	200	200–200	149·4	130·8–163·1	0·4	0·2–0·5	8·1	7·1–8·9	0·2	0·1–0·4	191·1	190·4–192·2
Pretzels	4	35	35–35	601·6	574·3–627·7	3·4	3·2–3·7	25·3	24·4–27·3	3·2	1·6–4·3	2·0	1·9–2·2
Wafers	4	89	81–94	1909·5	1788·7–2120·7	4·5	3·5–5·2	59·0	52·5–68·0	22·4	17·6–30·6	1·9	0·9–2·8
Home-made food													
*Cheburec*	2	117	98–137	1503·9	1175·3–1832·5	9·9	8·3–11·4	47·8	39·9–55·8	14·3	9·8–18·8	43·5	38·7–48·3
*Pateuri*	5	129	58–293	1588·5	714·7–3591·3	9·6	4·2–14·2	44·0	26·5–90·6	18·3	3·6–48·8	54·7	19·4–134·7
Sausage roll	4	125	100–149	1322·8	1130·3–1686·9	11·9	9·9–13·5	40·9	29·9–59·7	11·7	10·8–12·7	57·5	42·5–76·7
Savoury *plăcintă*	5	118	90–157	1587·4	1174·7–2450·0	10·1	6·6–12·1	44·5	31·2–57·0	17·9	8·3–34·6	43·6	32·9–52·6
Sweet doughnut	4	49	34–61	760·9	533·8–967·6	2·7	1·8–3·9	21·2	14·7–29·9	9·6	6·9–14·0	14·8	10·9–18·2
Sweet *plăcintă*	4	101	82–143	1359·0	937·1–1661·7	4·9	3·1–5·8	52·9	40·6–68·7	10·4	5·5–13·2	31·9	18·5–56·6
Sweet roll (*chiflă*)	4	126	79–209	1809·2	1230·2–2538·1	7·1	5·2–9·6	61·4	41·8–91·6	17·6	11·8–22·4	37·7	18·6–82·7

Min, minimum; Max, maximum.

* To convert energy in kJ to kcal, the values should be divided by 4·184.

Regarding the lipid profile, the highest mean content in SFA and MUFA was found in industrial wafers (10·9 and 8·2 g/serving, respectively) and industrial chocolate and chocolate candy (8·1 and 7·4 g/serving, respectively). The highest content in PUFA, particularly *n*-6, was found in home-made savoury pastries such as *pateuri* (9·1 and 9·0 g/serving, respectively) and *plăcintă* (5·4 and 5·3 g/serving, respectively). *n*-3 Fatty acids were present in reduced amounts in most foods; the maximum value was found in home-made sausage roll (0·2 g/serving). Home-made sweet *chiflă* (1·4 g/serving) and savoury *plăcintă* (1·3 g/serving) presented the highest mean TFA content (Table 3).

**Table 3.** Nutritional composition (fatty acid profile) of the street food samples evaluated by laboratorial analysis, per serving (Mean values and ranges)

		Serving size (g/serving)		SFA (g/serving)		MUFA (g/serving)		PUFA (g/serving)		*n*-6 (g/serving)		*n*-3 (g/serving)		TFA (g/serving)	
	*n*	Mean	Min–max	Mean	Min–max	Mean	Min–max	Mean	Min–max	Mean	Min–max	Mean	Min–max	Mean	Min–max
Industrial food															
Biscuits	4	88	82–98	2·8	2·7–2·8	2·8	2·4–3·2	2·0	1·1–3·0	2·0	1·1−3·0	0·0	0·0–0·0	0·2	0·1–0·5
Chocolate	4	33	33–33	8·1	2·2–16·1	7·4	2·2–12·7	0·9	0·6–1·7	0·9	0·5–1·6	0·0	0·0–0·1	0·7	0·0–2·6
Ice cream	4	56	33–71	5·0	1·5–7·2	1·6	0·2–2·5	0·5	0·0–1·1	0·5	0·0–1·1	0·0	0·0–0·0	0·4	0·0–0·7
*Kvass*	4	200	200–200	0·0	0·0–0·1	0·1	0·0–0·1	0·1	0·0–0·1	0·1	0·0–0·1	0·0	0·0–0·0	0·0	0·0–0·1
Pretzels	4	35	35–35	0·5	0·2–0·6	0·8	0·4–1·1	1·9	1·0–2·8	1·9	0·9–2·7	0·0	0·0–0·0	0·1	0·0–0·0
Wafers	4	89	81–94	10·9	8·7–14·4	8·2	6·3–11·0	2·8	1·9–5·2	2·7	1·8–5·1	0·0	0·0–0·1	0·5	0·1–1·9
Home-made food															
*Cheburec*	2	117	98–137	4·9	1·6–8·1	4·7	3·2–6·1	4·1	3·4–4·9	4·0	3·2–4·8	0·1	0·1–0·1	0·6	0·0–1·2
*Pateuri*	5	129	58–293	3·2	0·9–7·4	5·7	1·2–15·5	9·1	1·4–25·6	9·0	1·4–25·3	0·1	0·0–0·2	0·2	0·1–0·4
Sausage roll	4	125	100–149	3·7	3·3–4·1	4·8	4·4–5·1	3·0	1·9–3·5	2·8	1·7–3·3	0·2	0·1–0·2	0·1	0·1–0·2
Savoury *plăcintă*	5	118	90–157	6·0	2·9–9·2	5·2	2·2–11·2	5·4	2·9–12·7	5·3	2·9–12·7	0·1	0·0–0·1	1·3	0·2–2·3
Sweet doughnut	4	49	34–61	1·4	1·1–2·1	3·0	2·1–4·3	5·1	3·6–7·4	5·0	3·6–7·4	0·0	0·0–0·0	0·1	0·1–0·2
Sweet *plăcintă*	4	101	82–143	3·2	0·8–5·4	2·9	1·5–3·6	3·3	1·9–6·0	3·3	1·9–6·0	0·0	0·0–0·0	1·0	0·1–2·0
Sweet roll (*chiflă*)	4	126	79–209	7·5	5·4–10·1	5·3	3·7–7·1	3·3	1·9–5·2	3·3	1·8–5·1	0·1	0·0–0·1	1·4	0·8–2·7

Min, minimum; Max, maximum; TFA, *trans*-fatty acids.

Mean Na content ranged between 2 mg/serving in industrial chocolate and chocolate candy and 774 mg/serving in home-made sweet *chiflă*, whereas mean K content ranged between 12 mg/serving in industrial *kvass* and 216 mg/serving in industrial chocolate. The average Na:K ratio was higher than 5 in most foods, reaching 8·7 in pretzels (Table 4).

**Table 4.** Nutritional composition (sodium, potassium and sodium:potassium ratio) of the street food samples evaluated by laboratorial analysis, per serving (Mean values and ranges)

		Serving size (g/serving)		Na (mg/serving)		K (mg/serving)		Na:K ratio	
	*n*	Mean	Min–max	Mean	Min–max	Mean	Min–max	Mean	Min–max
Industrial food									
Biscuits	4	88	82–98	57	51–63	55	31–68	2·0	1·3–3·4
Chocolate	4	33	33–33	2	0–8	216	135–298	0·0	0·0–0·1
Ice cream	4	56	33–71	30	15–46	80	67–108	0·6	0·4–0·9
*Kvass*	4	200	200–200	22	19–28	14	13–15	2·8	2·5–3·1
Pretzels	4	35	35–35	285	135–640	57	45–71	8·7	4·3–19·9
Wafers	4	89	81–94	127	73–188	109	46–162	2·1	1·5–2·7
Home-made food									
*Cheburec*	2	117	98–137	474	383–566	152	127–178	5·3	5·1–5·4
*Pateuri*	5	129	58–293	447	201–1119	175	32–583	8·3	3·2–25·2
Sausage roll	4	125	100–149	630	486–803	158	106–190	7·0	4·6–9·6
Savoury *plăcintă*	5	118	90–157	528	338–1029	154	98–198	5·9	3·5–10·5
Sweet doughnut	4	49	34–61	100	52–146	29	17–38	5·6	4·2–6·7
Sweet *plăcintă*	4	101	82–143	186	107–357	63	40–115	6·4	2·4–15·0
Sweet roll (*chiflă*)	4	126	79–209	774	396–1533	212	112–338	6·1	4·5–7·7

Min, minimum; Max, maximum.

## Discussion

Street food vending sites selling food and beverages were highly frequent in Chişinău. Mostly industrial, but also home-made items were available, including a range of energy-dense confectionery, sweet and savoury snacks and pastries, high in SFA, TFA and Na.

This urban street food environment has particular characteristics. A higher proportion of stationary vending sites, mostly formal such as stands and kiosks, was found in Chişinău, in contrast with other urban settings in lower-middle-income country^(11,25,26)^. Also, the street foods available were more frequently westernised food options (e.g. industrial snacks, confectionery and soft drinks), resembling characteristics of the nutrition transition occurring in Eastern Europe^(1–3)^. These foods, sugar-rich and energy-dense, may contribute to weight gain^(27)^, mainly when presented in considerable serving sizes. Furthermore, alcoholic drinks were widely available, an additional source of public health concern, given their high consumption in the country and a strong association with the burden of NCD^(28)^.

Home-made and industrial foods were found to have medium (12·6 kJ/g) and high (18·0 kJ/g) mean energy density, respectively^(29)^, which corroborates previous findings that street foods are energy-dense sources of nutrients for populations worldwide^(10)^. Industrial wafers and biscuits presented the highest energy density (approximately 20·9 kJ/g), possibly reflecting a high sugar and fat content^(30)^, as previously observed in samples of street food in Dushanbe, Tajikistan^(26)^. Although the free sugar content was not discriminated in this study, they may have a high sugar content, which is line with existing literature on street food composition^(10,25,26,31)^.

Industrial wafers and home-made savoury pastries (e.g. *pateuri* and *placintă*) presented the highest fat content, approximately half of their total energy. Both industrial and home-made foods were found to be essential sources of SFA and TFA. Almost 25 % of the energy of industrial wafers and 16 % of the energy of some home-made sweet pastries came from SFA. For some home-made pastries, the contribution of TFA for the total energy reached approximately 3 %. One serving encompassed over half the daily WHO recommendation for TFA (<1 % total energy value/d), considering an average adult daily intake of 8368 kJ^(32)^ and exceeded the limit of 2 g TFA/100 g fat, defined by the Eurasian Economic Union^(33)^ and the European Union^(34)^, with some food samples reaching 123 % of the limit. The considerable variation in the SFA and TFA content of such home-made pastries may be due to the use of different cooking methods or to the type and quantity of the ingredients used^(10,12)^, particularly the fat sources^(35)^. The use of butter, even if in mixture with other fats, cannot be excluded, as it is a natural source of TFA, with a different relevance from industrially produced by hydrogenation.

The primary sources of protein were home-made savoury pastries, mostly meat-based, in line with the reported trend of high consumption of red and processed meat in Eastern Europe^(3,10,25)^. These were also the primary Na sources, with one serving supplying, on average, 5·1 to 38·7 % of the maximum daily recommended intake (100–774 mg)^(36)^. These findings concur with national estimates that one serving of savoury pastries contained, on average, 512 mg of Na. This study also revealed that some sweet pastries contained a similar content, meaning that this nutrient may be hidden in foods where the consumer would not expect, as found in central Asian countries^(37)^. Most foods do not comply with the WHO recommendation of Na:K ratio below 1^(36)^. In addition, fruit and vegetables, rich in K, were infrequently observed in this street food environment. These findings reinforce concerns about the low consumption of these foods, and unbalanced Na/K intake among the Moldovan population, markedly in urban settings^(38,39)^. Increasing the accessibility to these food options in urban Moldova could counterbalance the broader availability of unhealthier foods options, leading to better food choices among consumers^(40)^.

Street food products are available for consumption throughout the year, which is an opportunity for policy intervention. The improvement of the nutritional profile of the available street foods might be integrated in the National Strategy for Prevention and Control of NCD, which aims the shift towards healthy diets^(38)^, following recent international guidelines on sustainable food systems^(41)^. First, legislative approaches in cooperation with the food industry might be useful for effective salt reduction and TFA elimination, possibly through setting maximum limits in foods, food labelling or product reformulation. Additionally, given the central economic role of agriculture^(42)^, investing in local and sustainable food production would promote the availability of fresh and affordable fruits and vegetables in local markets. As evidenced in contexts where street food is part of the tradition^(43)^, addressing nutrition education actions for the general population and vendors would ultimately increase public knowledge and skills to create healthier recipes while maintaining the cultural identity. The future study of vendors’ and consumers’ nutritional literacy may contribute to design nutritional education strategies specific to their needs^(44)^.

### Strengths and limitations

One of the strengths of this study is the fact that it was conducted in a region where the street food environment has been understudied^(14)^, adding insight into the characteristics of more urbanised settings. The stepwise approach for data collection and analysis aimed to ensure an unbiased and comprehensive characterisation of the street food environment. Although the results may not be generalised to other communities due to local cultural specificities, the methodology has a vast potential to be adapted to different settings^(17)^, allowing comparison of results. The low proportion of fruit found in Chişinău is in line with findings in urban Tajikistan^(26)^; it should be noted that vending sites selling exclusively unprepared fresh fruit were not considered eligible in the FEEDcities study because these are usually more oriented to sell for household consumption and not for immediate consumption, as the definition of ‘ready-to-eat’ implies. The study was conducted in the summer, and the observed number and variability of types of vending sites might be higher than in other seasons, given that a substantial proportion of the informal vendors reported not working under adverse weather conditions. Additionally, as described in less urbanised settings, street foods may vary according to the agricultural seasonality^(11)^. Nevertheless, we expect that in this urban area seasonality might affect mostly the availability of some ingredients used in the preparation of home-made street foods (e.g. fillings of sweet and savoury pastries), but not the availability of major food groups, neither their nutritional composition in terms of sugar, salt and fat. The routine implementation of this methodology would be an interesting tool to monitor several characteristics of the street food environment^(45)^ while minimising the issue of seasonal variability. The nutritional composition of the analysed street foods was estimated by chemical analysis, using reliable methodologies, which overcomes limitations of previous studies^(3,10,14)^.

### Conclusion

In Chişinău, the street foods available include westernised options, coexisting with traditional home-made snacks and pastries, some of which very energy-dense and served in considerable serving sizes. It is imperative to improve the nutritional profile of street food while protecting its cultural and community role. Increasing fruit and vegetable availability, reducing salt content and encouraging the use of healthier fats is crucial to prevent diet-related NCD.

## Supplementary material

For supplementary material accompanying this paper visit https://doi.org/10.1017/S0007114520002020. click here to view supplementary material
