# Supplementary material for: Street food in Eastern Europe: a perspective from an urban environment in Moldova
Source: Br J Nutr. 2020 Jun 9;124(10):1093–101. doi: 10.1017/S0007114520002020 (PMC7547890; doi:10.1017/S0007114520002020)
Supplement: Supplementary file 1 [file S0007114520002020sup001.docx]

**Street food in Eastern Europe: a perspective from an urban environment in Moldova**

**Supplementary Material**

| **Supplementary Table S1. Characteristics of participants and non-participants (n=439)** | | | | | | |
| --- | --- | --- | --- | --- | --- | --- |
|  | **Total (n=439)** | **Participation** | | | |  |
|  |  | **No**  **(n=111)** | | **Yes**  **(n=328)** | |  |
|  | **n** | **n** | **%** | **n** | **%** | **p** |
| **Location within the buffer** | |  |  |  |  | **<0.001^a^** |
| Inside the market | 237 | 79 | 71.2 | 158 | 48.2 |  |
| Surroundings | 202 | 32 | 28.8 | 170 | 51.8 |  |
| **Type of vending site** | |  |  |  |  | 1.000 |
| Stationary | 421 | 107 | 96.4 | 314 | 95.7 |  |
| Mobile | 18 | 4 | 3.6 | 14 | 4.3 |  |
| **Physical setup (stationary)** | |  |  |  |  | **<0.001^b^** |
| Stand | 170 | 37 | 34.6 | 133 | 42.4 |  |
| Kiosk | 99 | 30 | 28.0 | 69 | 22.0 |  |
| Freezer or soft ice cream machine | 46 | 14 | 13.1 | 32 | 10.2 |  |
| *Dukoni* | 43 | 6 | 5.6 | 37 | 11.8 |  |
| Bench with table | 23 | 14 | 13.1 | 9 | 2.9 |  |
| *Kvass* barrel | 29 | 4 | 3.7 | 25 | 8.0 |  |
| Truck | 8 | 0 | 0.0 | 8 | 2.6 |  |
| Pushcart | 1 | 1 | 0.9 | 0 | 0.0 |  |
| Table with chairs | 2 | 1 | 0.9 | 1 | 0.3 |  |
| ^a^ Statistically significant differences according to Pearson’s Chi-square test, for a confidence level of 95% (p-value<0.05)  ^a^ Statistically significant differences according to Fisher’s exact test, for a confidence level of 95% (p-value<0.05) | | | | | | |

| **Supplementary Table S2. Characteristics of the stationary street food vending sites by physical setups in Chișinău, Moldova (n=314)** | | | | | | | |
| --- | --- | --- | --- | --- | --- | --- | --- |
|  | **Total** | | **Physical Setups^a^** | | | |  |
| **Characteristics** |  |  | **Informal**  **(n=41)** | | **Formal (n=273)** | | **p** |
| **Food vendor** | **n** | **%** | **n** | **%** | **n** | **%** |  |
| Food vendor sex (women) | 280 | 89.2 | 37 | 90.2 | 243 | 89.0 | 0.813 |
| Food vendor ownership (owner) | 24 | 7.6 | 2 | 4.9 | 22 | 8.1 | 0.752 |
| **Business** |  |  |  |  |  |  |  |
| Operating the whole week | 274 | 87.3 | 38 | 92.7 | 236 | 86.5 | 0.264 |
| Operating the whole year | 251 | 79.9 | 12 | 29.3 | 239 | 87.6 | **<0.001**^b^ |
| Operating under every type of weather | 255 | 81.2 | 20 | 48.8 | 235 | 86.1 | **<0.001**^b^ |
| **Infrastructure** |  |  |  |  |  |  |  |
| Access to electricity | 279 | 88.9 | 38 | 92.7 | 241 | 88.3 | 0.595 |
| Access to drinking water | 303 | 96.5 | 39 | 95.1 | 264 | 96.7 | 0.642 |
| Access to toilet facility | 306 | 97.5 | 40 | 97.6 | 266 | 97.4 | 1.000 |
| ^a^ **Informal physical setups:** freezer or ice machine (n=32)**;** bench with table (n= 9). **Formal physical setups**: stand, stall or booth (n= 133); kiosk (n=69); *dukoni* (n=37); kvass barrel (n=25); truck (n=8) and tables with chairs for customers (n=1)  ^b^ Statistically significant differences according to Pearson’s Chi-square test, for a confidence level of 95% (p-value<0.05) | | | | | | | |

**Supplementary Table S3. Examples of street food vending sites in Chișinău, Moldova**

| **Type of vending site** | **Example** |
| --- | --- |
| **Mobile** |  |
| Street vendor carrying a bag with foods or beverages. | **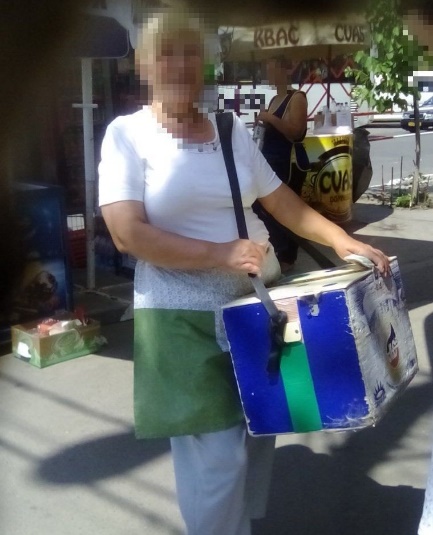** |
| **Stationary** |  |
| Stand  Upright structure where street food is prepared and/or displayed. | **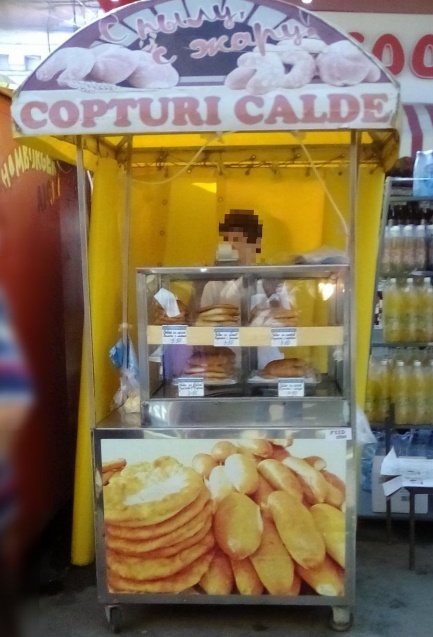** |
| Kiosk  Small open-fronted hut or cubicle where typically newspapers are sold, as well as a sort of foods and beverages. | **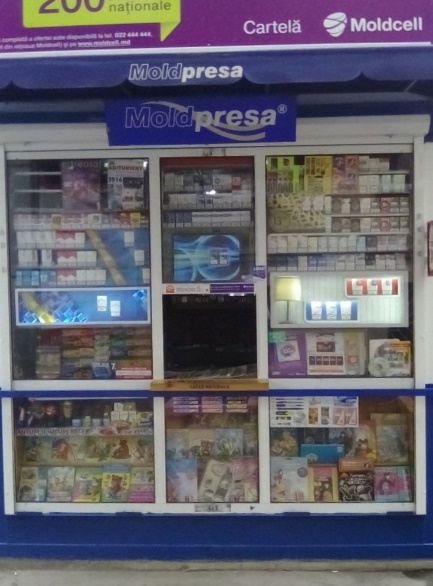** |
| Vending site consisting of freezer and refrigeration machines selling ice-creams and beverages. | **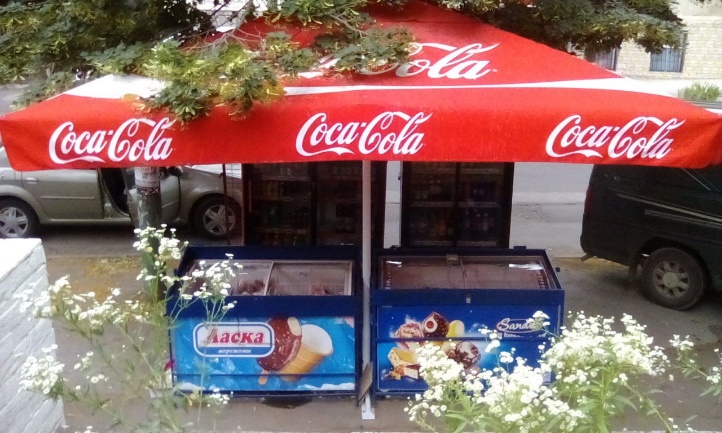** |
| *Dukoni*  Restaurant serving traditional fast food selling directly on the street through an open window. | **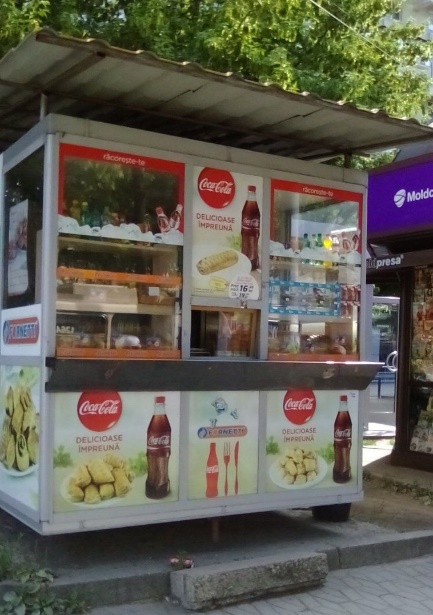** |
| *Kvass* barrel  Vending site consisting of a portable metal barrel containing *kvass (*a traditional fermented beverage made from rye bread), supported by an upright structure. | **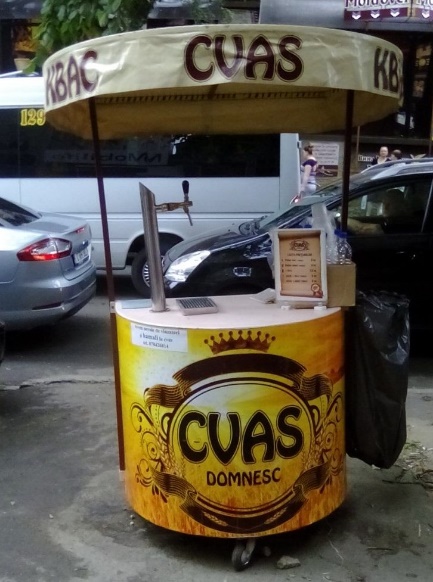** |
| Truck  Large motorized vehicle, such as a van or trailer, equipped to cook, prepare, serve, and/or sell food. | **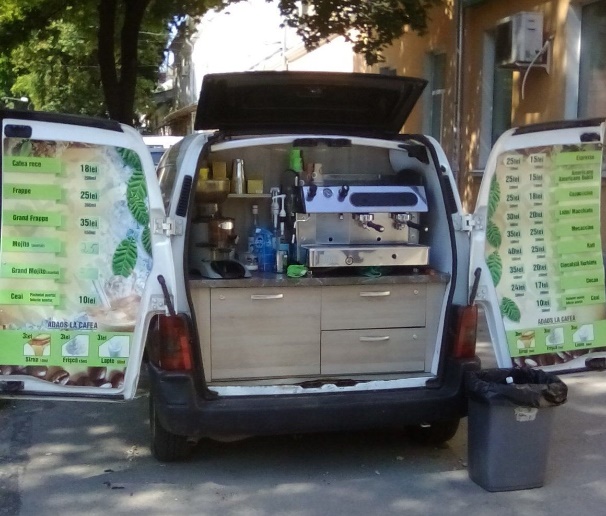** |
| No quality photos of “tables with chairs for customers” or “bench with tableboard” were registered. | |

**Supplementary Table S4. Examples of street foods collected in Chișinău, Moldova**

| **Street Food** | **Example** |
| --- | --- |
| **Industrial** | |
| Chocolate | 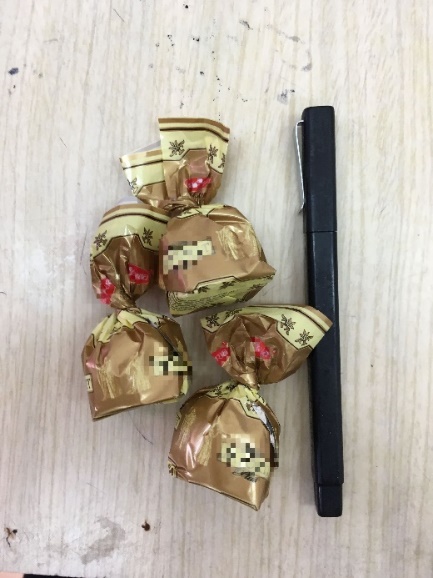 |
| Biscuits | 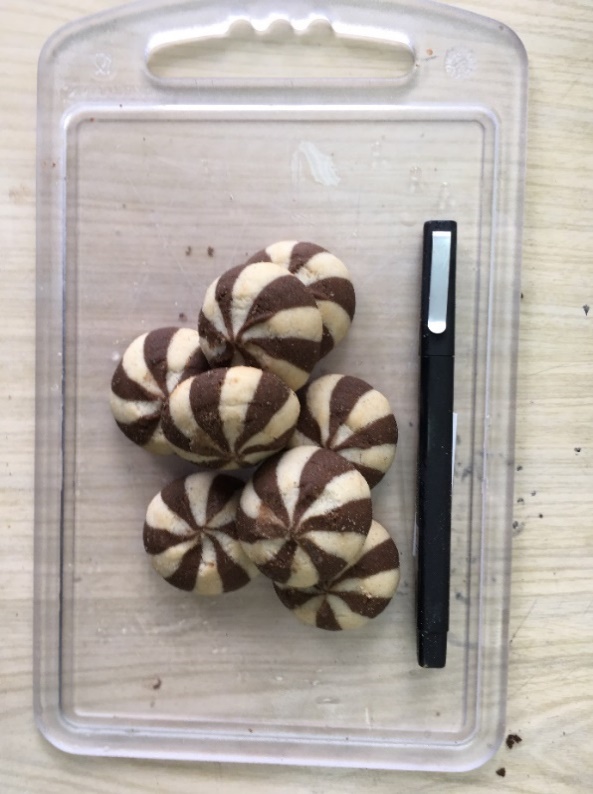 |
| Ice-cream | 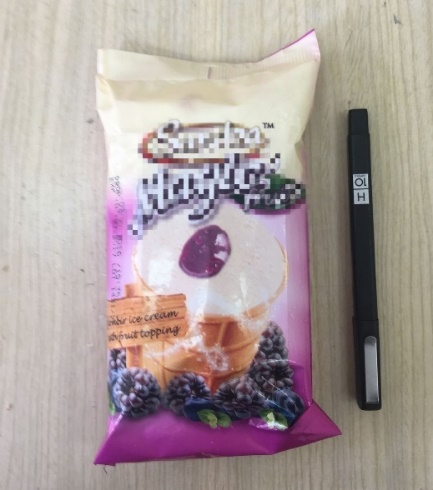 |
| *Kvass*:  traditional fermented beverage made from rye bread. | 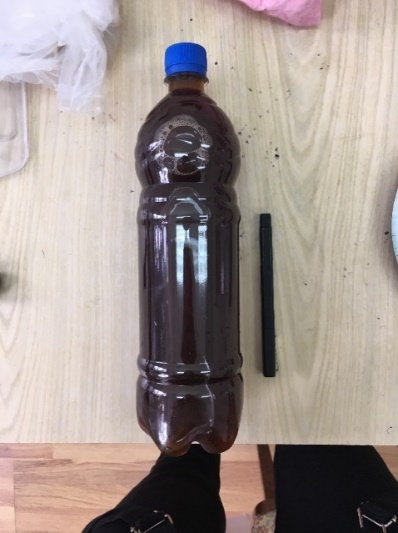 |
| Pretzels | 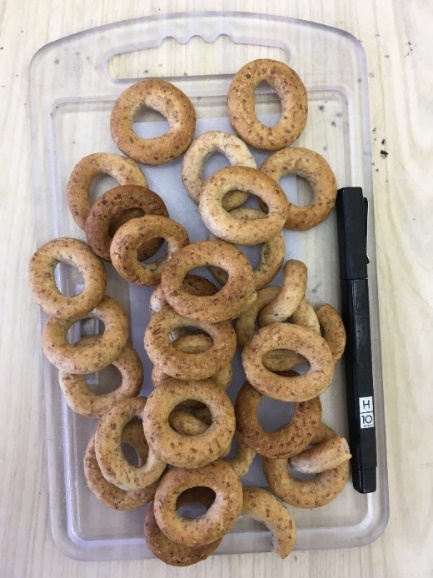 |
| Wafers | 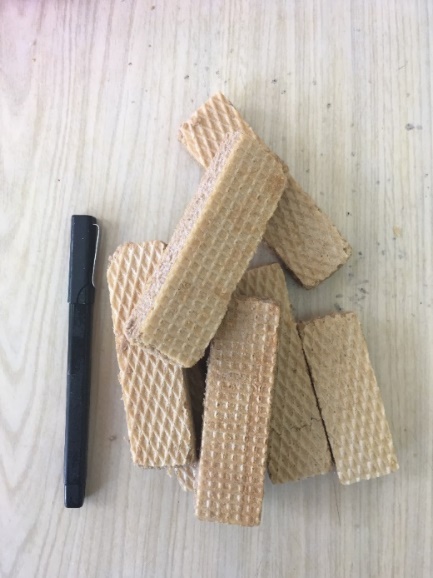 |
| **Homemade** | |
| *Cheburec*  Traditional fried savoury pastry generally filled with meat (e.g. chicken, beef). | 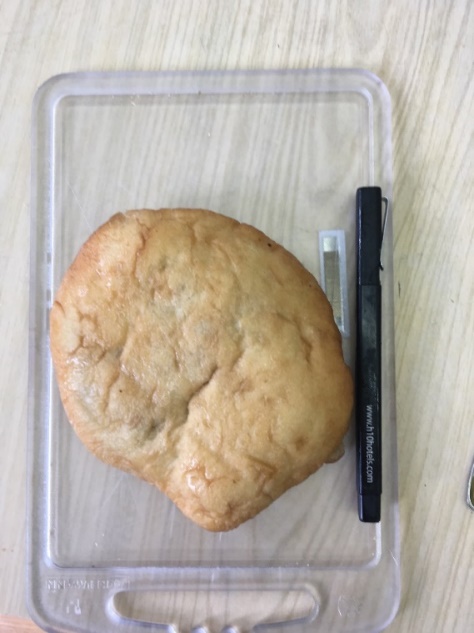 |
| Sweet roll (*chiflă)*  Sweet bun made of leavened dough (either flaky or brioche type), usually filled with a sweet filling such as chocolate, vanilla or cherry cream. | 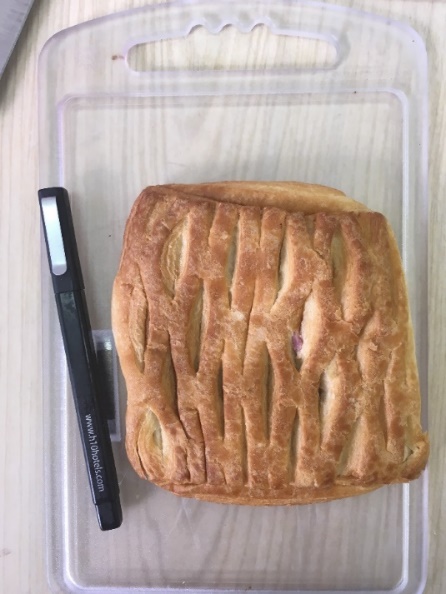 |
| Sausage roll or hot dog | 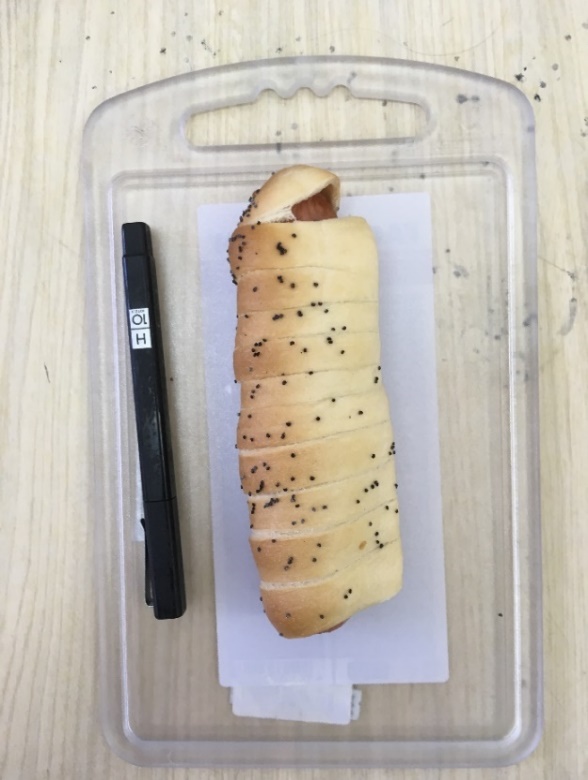 |
| *Pateuri* or *pateu*  Traditional fried pastry made from leavened dough filled with cheese, vegetables or meat, usually an oval shape and served in individual portions. | 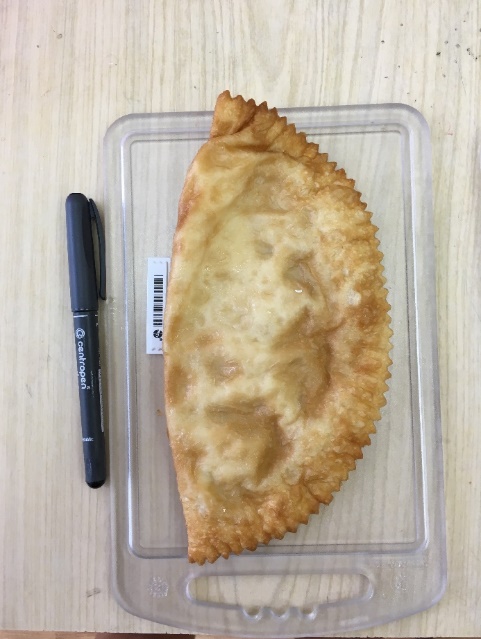 |
| *Plăcintă* (savoury)  Fried pastry, usually made from dough made of flour, water and sunflower oil, filled with cheese, vegetables, meat or potatoes or sweet fillings, usually served in a pie shape, but may have various shapes. | 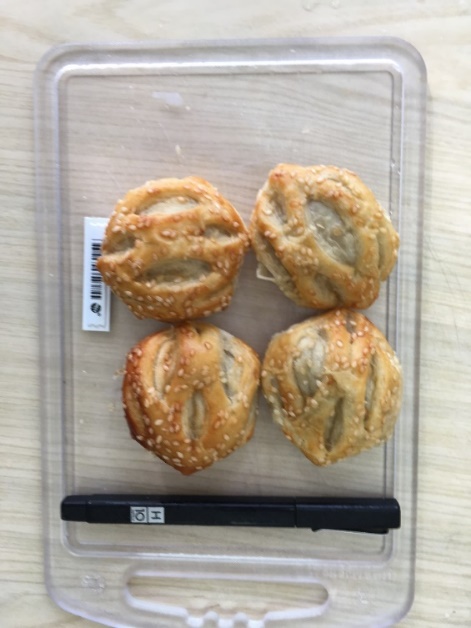 |
| *Plăcintă* (sweet):  Fried pastry, usually made from flour, water and sunflower oil, filled with a sweet filling as chocolate, vanilla or cherry cream. | 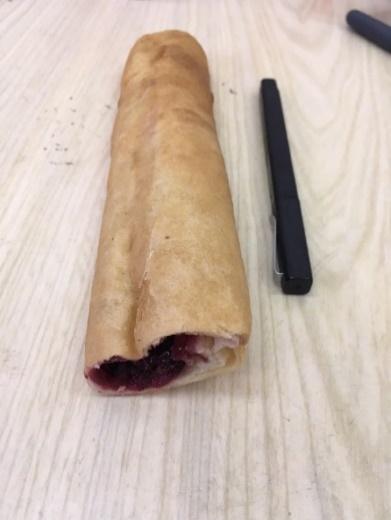 |
| Sweet doughnut | 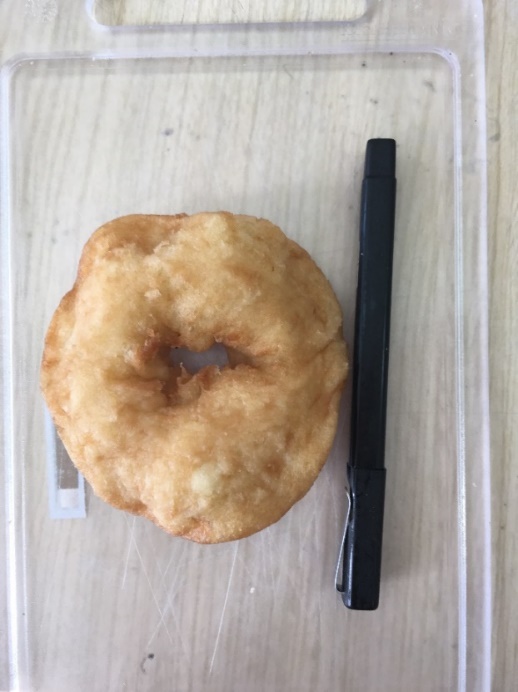 |


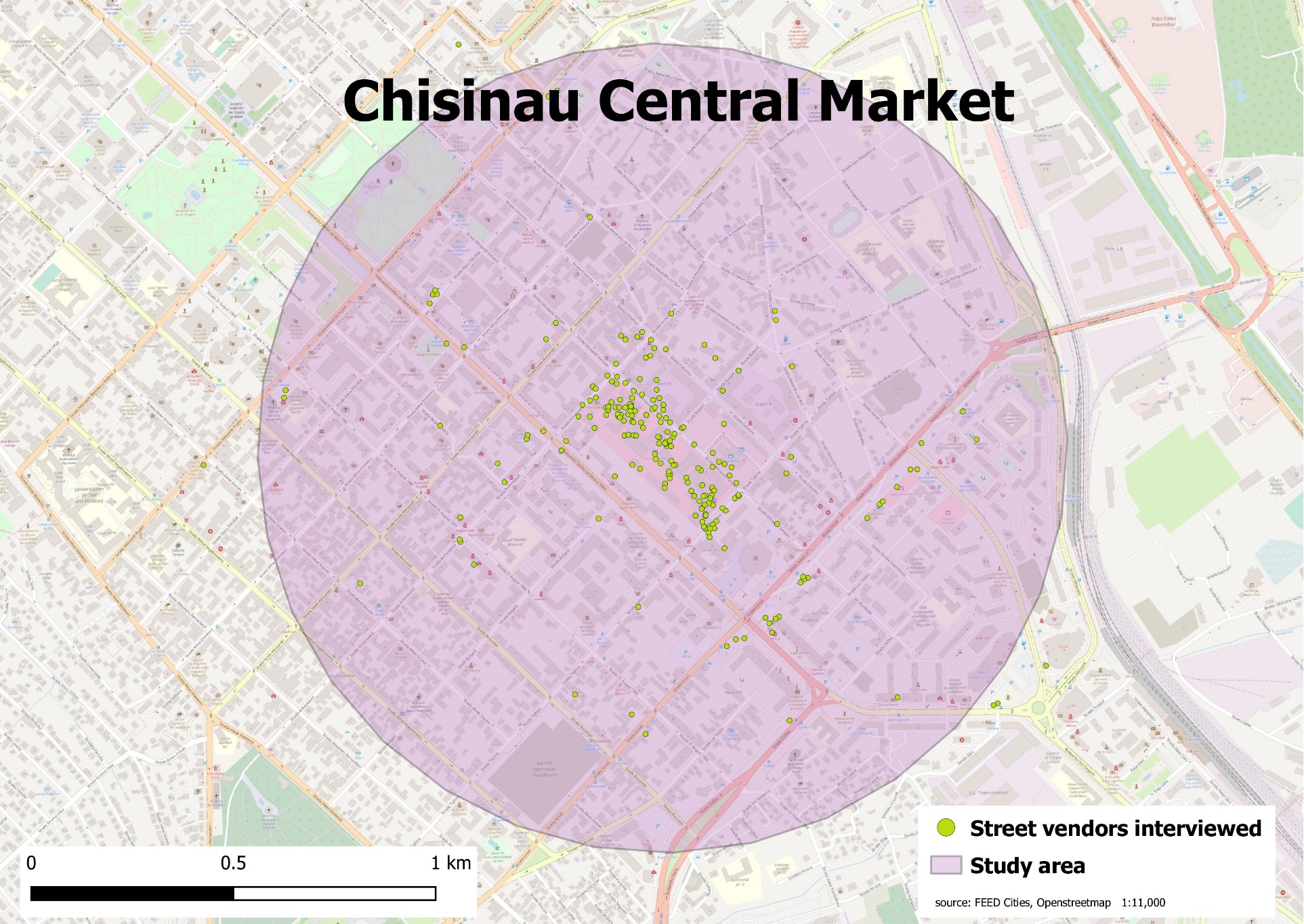


**Supplementary Figure 1. Geographical distribution of the street food vending sites in Chișinău, Moldova**
